# Enterohemorrhagic *Escherichia coli* infection inhibits colonic thiamin pyrophosphate uptake via transcriptional mechanism

**DOI:** 10.1371/journal.pone.0224234

**Published:** 2019-10-22

**Authors:** Kasin Yadunandam Anandam, Subrata Sabui, Morgan M. Thompson, Sreya Subramanian, Hamid M. Said

**Affiliations:** 1 University of California-Irvine School of Medicine, Department of Physiology and Biophysics, Irvine, California, United States of America; 2 Veterans Affairs Medical Center, Department of Medical Research, Long Beach, California, United States of America; 3 University of California-Irvine School of Medicine, Department of Medicine, Irvine, California, United States of America; University of Illinois at Chicago, UNITED STATES

## Abstract

Colonocytes possess a specific carrier-mediated uptake process for the microbiota-generated thiamin (vitamin B1) pyrophosphate (TPP) that involves the TPP transporter (TPPT; product of the *SLC44A4* gene). Little is known about the effect of exogenous factors (including enteric pathogens) on the colonic TPP uptake process. Our aim in this study was to investigate the effect of Enterohemorrhagic *Escherichia coli* (EHEC) infection on colonic uptake of TPP. We used human-derived colonic epithelial NCM460 cells and mice in our investigation. The results showed that infecting NCM460 cells with live EHEC (but not with heat-killed EHEC, EHEC culture supernatant, or with non-pathogenic *E*. *Coli*) to lead to a significant inhibition in carrier-mediated TPP uptake, as well as in level of expression of the TPPT protein and mRNA. Similarly, infecting mice with EHEC led to a significant inhibition in colonic TPP uptake and in level of expression of TPPT protein and mRNA. The inhibitory effect of EHEC on TPP uptake by NCM460 was found to be associated with reduction in the rate of transcription of the *SLC44A4* gene as indicated by the significant reduction in the activity of the *SLC44A4* promoter transfected into EHEC infected cells. The latter was also associated with a marked reduction in the level of expression of the transcription factors CREB-1 and ELF3, which are known to drive the activity of the *SLC44A4* promoter. Finally, blocking the ERK1/2 and NF-kB signaling pathways in NCM460 cells significantly reversed the level of EHEC inhibition in TPP uptake and TPPT expression. Collectively, these findings show, for the first time, that EHEC infection significantly inhibit colonic uptake of TPP, and that this effect appears to be exerted at the level of *SLC44A4* transcription and involves the ERK1/2 and NF-kB signaling pathways.

## Introduction

Thiamin pyrophosphate (TPP; also called thiamin di-phosphate), is a biologically active form of vitamin B1 that acts as a cofactor for multiple enzymes (pyruvate dehydrogenase, α-ketoglutarate dehydrogenase, branched-chain α-ketoacid dehydrogenase, transketolase) that are involved in critical metabolic reactions (e. g., energy metabolism, reduction of cellular oxidative stress) [1, 2, 3]. The vitamin also plays a role in maintaining normal mitochondrial function and structure [4], and in cellular pro-inflammatory responses [5, 6]. Deficiency of thiamin in humans leads to serious clinical abnormalities (that include cardiovascular and neurological disorders), and occurs in chronic alcoholism and diabetes mellitus among other conditions [7, 8, 9].

Human/other mammals lack the ability to synthesize thiamin endogenously; therefore, they must obtain the micronutrient from exogenous sources via intestinal absorption. The intestine encounters two sources of thiamin: dietary and bacterial sources (the latter is in reference to the vitamin that is generated by the gut microbiota). With regards to the dietary source, the vitamin exists mainly in the phosphorylated form; this form is hydrolyzed to free thiamin prior to absorption by the action of the abundant small intestinal phosphatases [10, 11, 12]. The liberated free thiamin is then absorbed via a specific carrier-mediated process that involves both the *SLC19A2* (THTR-1) and *SLC19A3* (THTR-2) transport systems [10–17]. As to the microbiota-generated vitamin B1, this source provides thiamin in both the free and the phosphorylated (i. e., TPP) forms [12, 18]. Studies from our laboratory have shown that the human/mammalian colonocytes are capable of absorbing both of these forms of the vitamin and that this occurs via distinct and specific carrier-mediated mechanisms [19–21]. Large intestinal absorption of free thiamin involves the *SLC19A2* and *SLC19A3* uptake systems [12, 19, 22], while that of TPP involves the recently identified TPPT system (product of the *SLC44A4* gene) [20, 21]. Other studies from our laboratory have shown that expression of the *SLC44A4* system in the gastrointestinal tract is restricted to the large intestine [21], and that this site-specific expression is determined by epigenetic mechanisms [23]. In addition, we have characterized different regulatory aspects of the TPPT system, identified a role for the cis-regulatory elements CREB-1 and ETS/ELF3 in basal activity of the *SLC44A4* promoter [24], and showed that the colonic TPP uptake process is adaptively-regulated by the prevailing extracellular substrate level [20, 25]. Very little, however, is known about the effect of external factors (including that of enteric pathogens) on the colonic TPP uptake process. In this study, we examined the effect of one such factor, i. e., infection with Enterohemorrhagic *E*. *coli* (EHEC), on colonic uptake of TPP. EHEC causes foodborne diarrhea in humans, and it mainly colonizes the colon and exert its effect via toxin -dependent and toxin-independent mechanisms [26, 27, 28]. We used the human-derived colonic epithelial NCM460 cells and mice for *in vitro* and *in vivo* models of infection, respectively. Our results showed that EHEC infection causes a significant inhibition in colonic TPP uptake and that this inhibition is exerted at the level of transcription of the *SLC44A4* gene and involves the ERK1/2 and NF-kB signaling pathways.

## Materials and methods

### Materials

NCM460 cells were from INCELL (San Antonio, TX), and [^3^H]-TPP (specific activity 1.3 Ci/mmol; radiochemical purity 97%) was from Moravek Biochemicals (Brea, CA); qPCR primers were from Sigma Genosys (Woodlands, TX); Other chemicals/reagents were from commercial vendors and were of analytical/molecular biology grade. Human and mouse specific anti-SLC44A4 polyclonal antibodies were generated for us by Alpha Diagnostics International (San Antonio, TX) and by Thermofisher (Rockford, IL), respectively. Human anti-ELF3 (AV31639) antibody was from Sigma-Aldrich (Saint Louis, MO); anti-CREB-1 (#9197), anti-ERK1/2 (#9102S), anti-Phospho ERK1/2 (#9101S) and anti-Phospho NF-κB (#3033S) were from Cell Signaling Technology (Danvers, MA); anti-NF-κB (ab16502) antibody from Abcam (Cambridge, MA); anti-β-actin (sc-47778) and anti-Lamin B (sc-6216) antibodies were from Santa Cruz Biotechnology (Santa Cruz, CA). The secondary antibodies, anti-rabbit IRDye-800 and anti-mouse IRDye-680, were purchased from LI-COR Bioscience (Lincoln, NE).

### Cell culture

The human-derived colonic non-transformed NCM460 epithelial cells were routinely maintained in Ham’s F-12 culture medium supplemented with 20% (vol/vol) FBS and antibiotics at 37° C in a 5% CO_2_ environment. Confluent NCM460 cell monolayers were used in the physiological and molecular investigations.

### Bacterial culture and infection of NCM460 cells

Wild-type EHEC (EDL933), as well as the EHEC mutants EHEC Δtir, EHEC ΔespF and EHEC ΔescN (all were kindly provided to us by Dr. G Hecht, of Loyola University Chicago, IL), and the non-pathogenic *E*. *coli* HS4 strain were used in this study. Overnight bacterial cultures were diluted to 1:50 in Luria-Bertani (LB) broth (EHEC ΔespF and EHEC ΔescN were grown in presence of 50 μg/ml kanamycin) and allowed to grow for 3 h to reach the exponential phase. Bacteria were resuspended in serum free media at the specified multiplicity of infection (MOI). Confluent NCM460 cells maintained in serum free media were infected with 200 MOI of wild-type EHEC and their mutants for 5 h [29, 30], then treated with gentamycin (50 μg/ml) for 60 min (to remove adherent EHEC), followed by utilization in the different studies. To study the effect of heat-killed (boiled) EHEC (200 MOI) on NCM460 cells, the bacteria was boiled for 30 min at 100° C.

### Bacterial culture supernatants

To collect bacterial culture supernatant, the overnight grown EHEC culture was centrifuged for 15 mins at 3000 rpm, followed by filtration (utilizing 0.22 μm sterile syringe filters) as described previously [31, 32].

### [^3^H]-TPP uptake studies *in vitro* and *in vivo*

Initial rate of carrier-mediated uptake (3 min at 37° C) of TPP [^3^H]-TPP (0.3 μCi/ml) [20, 21] was assayed in EHEC infected NCM460 cells (*in vitro*) using Krebs-Ringer (KR) buffer containing (in mM): 123 NaCl, 4.93 KCl, 1.23 MgSO_4_, 0.85 CaCl_2_, 5 glucose, 5 glutamine, 10 HEPES, and 10 MES (pH 7.4). Uptake reaction was terminated by the addition of ice-cold KR buffer followed by cell lysis (with 1 ml of 1 N NaOH), neutralization with 10 N HCl and radioactive counting in a liquid scintillation counter. Protein content of cell digests was measured using the Dc protein assay kit (Bio-Rad, Hercules, CA). For *in vivo* TPP uptake, eight-weeks-old C57BL/6J mice (~ 25 g) were given water containing streptomycin (5 g/L) for 24 h to eliminate the commensal microflora. The water was then replaced with regular water for another 24 h before bacterial infection. Mice were gavaged with 2x10^8^ CFU/mice (in 200 μl) [33] using 20-gauge gavage needle. Control (uninfected) mice were gavaged with same volume of PBS. After 72 h of infection, mice were euthanized and the colon sheet (~ 1 cm) were incubated *in vitro* in KR buffer containing [^3^H]-TPP in the presence or absence of unlabeled 1mM TPP for 10 minutes and then processed for radioactivity measurement. The animal protocol used in this study was approved by the Institutional Animal Care and Use Committee (IACUC), University of California, Irvine, CA.

### Quantitative real-time PCR analysis (RT-qPCR)

Total RNA isolated from NCM460 cells, and mouse colon infected with EHEC, were reverse transcribed (using iScript cDNA synthesis kit from Bio-Rad; Hercules, CA). Human and mouse gene specific primers (Table 1) were used to amplify the appropriate genes and PCR conditions were used as described previously [34]. Data was normalized to β-actin were quantified using a relative relationship method supplied by the iCycler manufacturer (Bio-Rad).

**Table 1:** Primers used for real-time PCR analysis.

Gene Name	Forward and Reverse Primers (5′– 3′)
hSLC44A4	TGCTGATGCTCATCTTCCTGCG; GGACAAAGGTGACCAGTGGGTA
hCREB-1	TTAACCATGACCAATGCAGCA; TGGTATGTTTGTACGTCTCCAGA
hELF3	TCTTCCCCAGCGATGGTTTTC; TCCCGGATGAACTCCCACA
h-β-actin	CATCCTGCGTCTGGACCT; TAATGTCACGCACGATTTCC
mSlc44a4	TGCCTACCAGAGTGTGAAGGAG; TGGCTTCCTTCAGCAGAGCGAT
m-β-actin	ATCCTCTTCCTCCCTGGA; TTCATGGATGCCACAGGA

### Transfection and firefly luciferase assay

*SLC44A4* wild-type (WT) promoter construct (3 μg/ml), along with 100 ng of *Renilla* luciferase-thymidine kinase (pRL-TK) plasmid (Promega, Madison, WI), were transiently transfected in NCM460 cells using Lipofectamine 2000 reagent (Life Technologies) for 24 h. Cells were subsequently infected with EHEC, then (5 h later) washed with gentamycin then lysed. The *Renilla*-normalized firefly luciferase activity was determined using a dual-luciferase assay system (Promega).

### Isolation of protein and western blot analysis

EHEC infected and uninfected NCM460 cells and mouse colon tissues were lysed in radio immunoprecipitation assay buffer (RIPA buffer; Sigma) containing complete protease inhibitor cocktail (Roche, NJ). The soluble protein fraction was collected followed by centrifugation (12,000 rpm, 20 min). Concentrations of proteins were determined using Dc protein assay kit (Bio-Rad). To determine the level of TPP transporter and transcription factors protein expression, an equal amount of protein (40 μg) was loaded onto 4–12% Bis-Tris gradient minigels (Invitrogen) and transferred in to Immobilon polyvinylidene difluoride membrane (Fisher Scientific). Subsequently, the blots were probed with anti-TPPT (1:200), anti-CREB-1 (1:200), anti-ELF3 (1: 200), anti-ERK1/2 (1:1000), anti-Phospho ERK1/2 (1:1000), anti-Phospho NF-κB (1:1000), anti-NF-κB (1:1000), anti-β-actin (1:3000), and anti-Lamin B (1: 1000) antibodies. The membranes were probed with anti-mTPPT antibody and anti-mTPPT antibody pretreated with antigenic peptide to show the specificity of the mTPPT antibody. Anti-rabbit IRDye-800 and anti-mouse IRDye-680 (both at 1:30,000 dilutions) were used as the secondary antibodies in this study. The specific immunoreactive bands were detected using the Odyssey infrared imaging system (LI-COR Bioscience, Lincoln, NE), and their densities were measured using the LI-COR software.

### Treatment of NCM460 cells with inhibitors

The ERK1/2 pathway specific inhibitor PD98059 (Peprotech, Rocky Hill, NJ) and the NF-kB pathway specific inhibitor Celastrol (Invivogen, San Diego, CA) were stored at -20° C. Prior to EHEC infection, the NCM460 cells were incubated with either PD98059 (50 μM) or Celastrol (100 nM) for 1h. The inhibitors were kept during the 5 h of infection.

### Statistical analysis

Uptake data presented in this study represent mean ± SE of 3 independent experiments (done on different occasions) and are expressed as percentage relative to simultaneously performed controls. The RT-qPCR, western blotting, and firefly luciferase assays were determined from three independent samples preparations. For statistical analysis, we used the Student’s t-test and P < 0.05 was considered to be statistically significant.

## Results

### Effect of EHEC on physiological/molecular parameters of the colonic TPP uptake process

#### *In vitro* studies using human-derived colonic epithelial NCM460 cells

**Fig 1:**
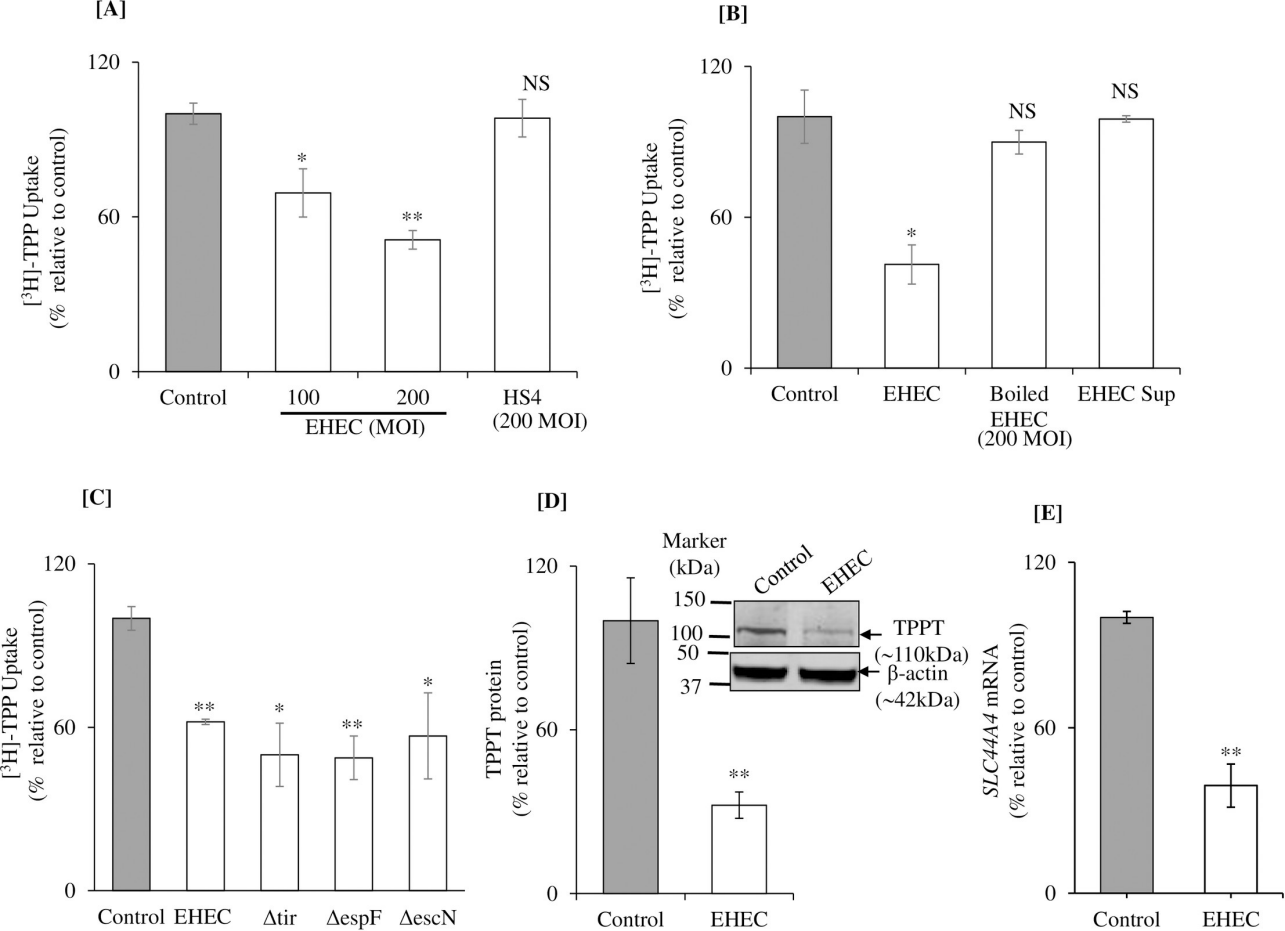
Effect of EHEC infection of human colonic epithelial NCM460 cells *in vitro* on carrier-mediated TPP uptake and on level of expression of TPP transporter (TPPT). **[A]** Carrier-mediated [^3^H]-TPP uptake was examined in NCM460 cells infected with EHEC at 100 and 200 MOI for 5 h. **[B]** Carrier-mediated TPP uptake was examined in NCM460 cells exposed to boiled (heat-killed) EHEC or bacterial culture supernatant. **[C]** Effect of infection with EHEC mutants (Δtir-EHEC, ΔespF-EHEC and ΔescN-EHEC) on [^3^H]-TPP uptake by NCM460 cells. **[D]** Levels of TPPT protein expression was examined in NCM460 cells infected with EHEC at 200 MOI for 5 h (by western blotting). Data were normalized with β-actin. **[E]** Levels of *SLC44A4* mRNA expression was examined in NCM460 cells infected with EHEC at 200 MOI for 5h (by RT-qPCR). Data are mean ± SE of 3–4 sets of independent experiments. **P < 0.01; * P < 0.05; NS-not significant.

We examined the effect of EHEC infection (using different multiplicity of infection, MOI), as well as that of the non-pathogenic *E*. *coli* HS4 (200 MOI), of colonic epithelial NCM460 cells on TPP uptake. For this, cells were exposed to EHEC for 5 h [29, 30], followed by evaluation of initial rate of carrier-mediated [^3^H]-TPP (0.23 μM) uptake [20]. The result showed a significant (P < 0.05 for 100 MOI, and P < 0.01 for 200 MOI) inhibition in TPP uptake by NCM460 cells infected with EHEC (but not with non-pathogenic *E*. *coli* HS4) compared with simultaneously processed uninfected controls (Fig 1A). A 200 MOI was used for all subsequent experiments. In another study, we investigated the effect of treating NCM460 cells with boiled EHEC (heat-killed bacteria) on TPP uptake with the results showing lack of effect on the vitamin uptake (Fig 1B). To assess whether the inhibition in TPP uptake caused by live EHEC is mediated via a secreted factor (toxin), we exposed the NCM460 cells to supernatant of culture EHEC (see “Methods”), then examined [^3^H]-TPP uptake. The result showed lack of inhibition of such a treatment on TPP uptake (Fig 1B).

EHEC causes attaching and effacing lesion via type III secretion system (T3SS), the molecular syringe that injects effector proteins into host cells. Thus, we examined functional involvement of EHEC effector proteins in the EHEC-induced inhibition in TPP uptake by NCM460 cells. For this, we used 200 MOI of EHEC Δtir (the translocated Intimin receptor facilitates the intestinal colonization), EHEC ΔespF (a multifunctional protein mainly involved in EHEC colonization) and EHEC ΔescN (putative ATPase component involved in the formation of T3SS and intestinal colonization) [29, 35] to infect NCM460 cells. Results showed a significant inhibition in TPP uptake by NCM460 cells infected with wild-type EHEC and their mutants, suggesting that these effector proteins are not required for the EHEC induced inhibition TPP uptake inhibition (Fig 1C).

In another study, we examined the effect of infecting NCM460 cells with EHEC (200 MOI; 5 h exposure) on level of TPPT protein expression. This was done by means of western blotting using specific polyclonal antibodies against the TPP transporter [25]. The result showed a significant (P < 0.01) reduction in the level of expression of the TPPT protein in cells infected with EHEC compared to uninfected controls (Fig 1D). We also examined (by mean RT-qPCR) the effect of infecting the NCM460 cells with EHEC on level of *SLC44A4* mRNA expression. The result showed a significant (P < 0.01) reduction in the level of expression of *SLC44A4* mRNA in cells infected with EHEC compared to uninfected cells (Fig 1E).

#### *In vivo* studies in mice

**Fig 2:**
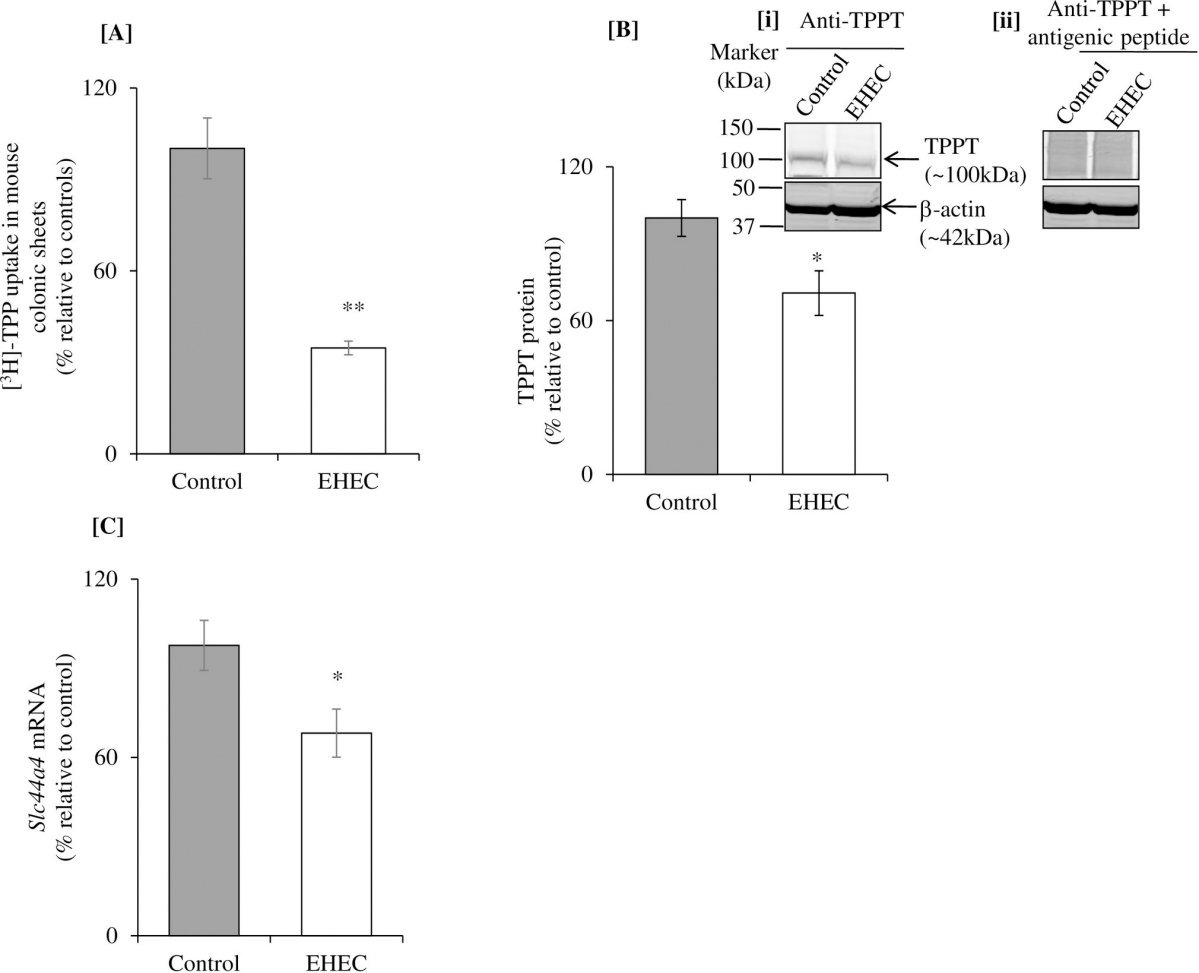
Effect of EHEC infection of mice *in vivo* on colonic carrier-mediated TPP uptake and on level of expression of TPPT. **[A]** Carrier-mediated [^3^H]-TPP uptake was examined in colonic sheet of C57BL/6J male mice (8–10 week-old) infected with EHEC for 72 h. **[B]** Levels of TPPT protein expression was examined in EHEC infected mouse colonic tissue. The membranes were incubated with custom made anti-TPPT antibodies [i] and anti-TPPT antibodies pretreated with antigenic peptide [ii]. The antigenic peptide was used to show the specificity of the TPPT. **[C]** Levels of *Slc44a4* mRNA expression was examined in EHEC infected colonic tissue. Data are mean ± SE of 3–4 pairs of mice. **P < 0.01; *P < 0.05.

In these studies, we investigated the effect of EHEC infection of mice *in vivo* on physiological/molecular parameters of the colonic TPP uptake process. For this, we gavaged C57BL/6J mice with EHEC (2x10^8^ CFU/mice; [33]), then examined TPP uptake 72 h following infection. The result showed a significant (P < 0.01) inhibition in colonic carrier-mediated TPP uptake (Fig 2A). We also examined (by western blot analysis) the level of TPPT protein expression in colonic cells of the infected mice and observed a significant (P < 0.01) reduction in mice infected with EHEC compared to uninfected controls (Fig 2B). Similarly, level of *Slc44a4* mRNA expression (determined by RT-qPCR) was found to be significantly (P < 0.05) reduced in EHEC infected mice compared to uninfected control (Fig 2C).

### Involvement of transcriptional mechanism in the effect of EHEC on colonic TPP uptake process

**Fig 3:**
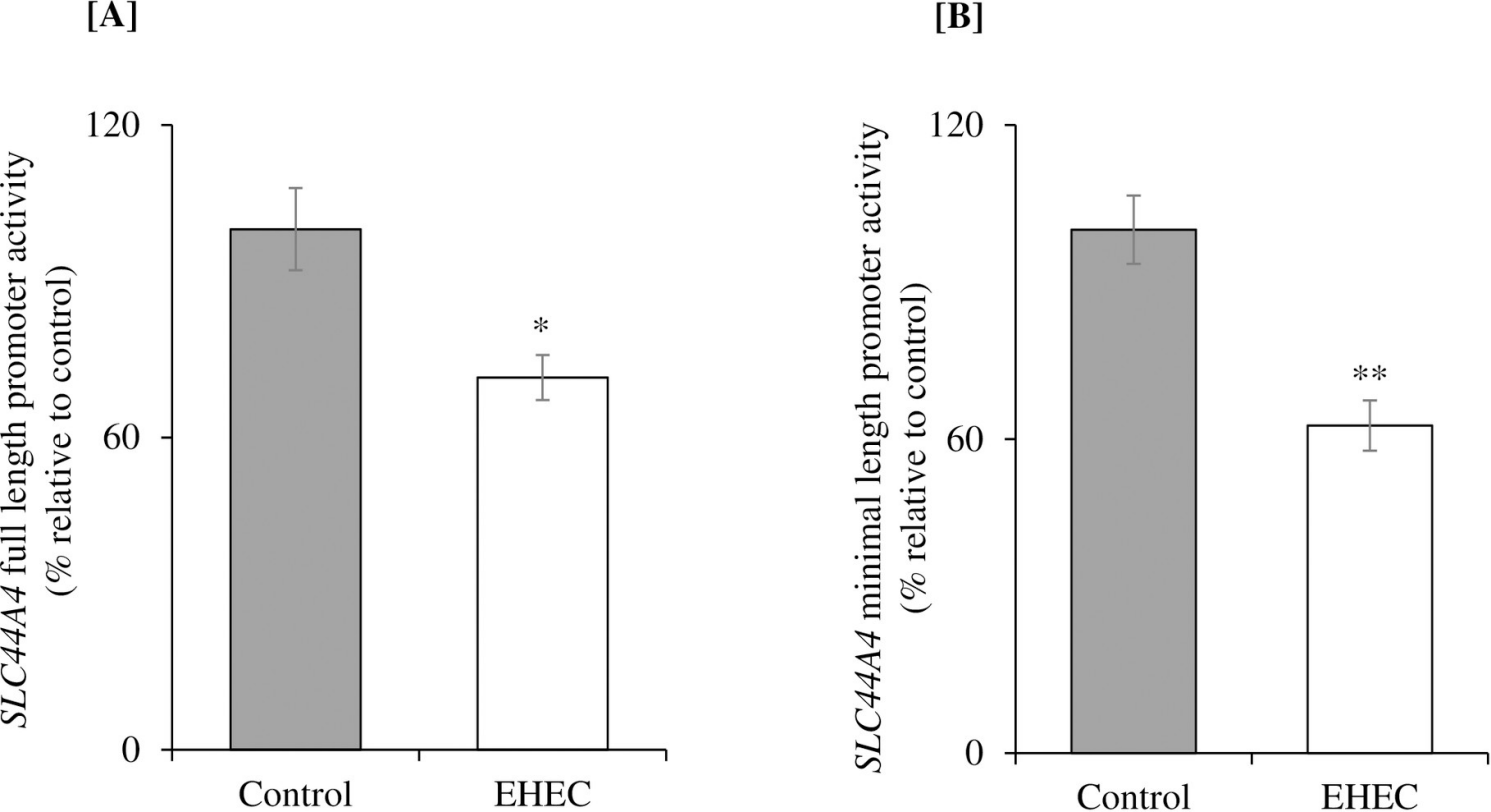
Effect of EHEC infection of NCM460 cells on activity of the *SLC44A4* promoter. The relative promoter activity of human *SLC44A4* full-length **[A]** and minimal promoter **[B]** constructs in pGL3 basic was examined in NCM460 cells infected with EHEC (200 MOI for 5 h) by luciferase assay. The relative values of promoter activity are expressed as fold change over pGL3 basic vector and data are presented as mean ± SE of 4 sets of independent experiments. **P < 0.01; *P < 0.05.

A change in level of expression of a given mRNA could be induced via multiple mechanisms including changes in the rate of transcription of the involved gene. To determine possible involvement of the latter mechanism in the observed effect of EHEC infection on expression of the colonic *SLC44A4* mRNA, we examined the effect of EHEC infecting of NCM460 cells expressing the full-length (and minimal-length) *SLC44A4* promoters (fused to the luciferase reporter) on activity of these promoters. The results showed that a significant inhibition in the activity of the *SLC44A4* full-length (P < 0.05) as well as the minimal (P < 0.01) promoters constructs in cells infected with EHEC compared to uninfected controls (Fig 3A & 3B). These results demonstrated that the effect of EHEC infection on *SLC44A4* expression is, at least in part, mediated at the level of transcription of the *SLC44A4* gene.

**Fig 4:**
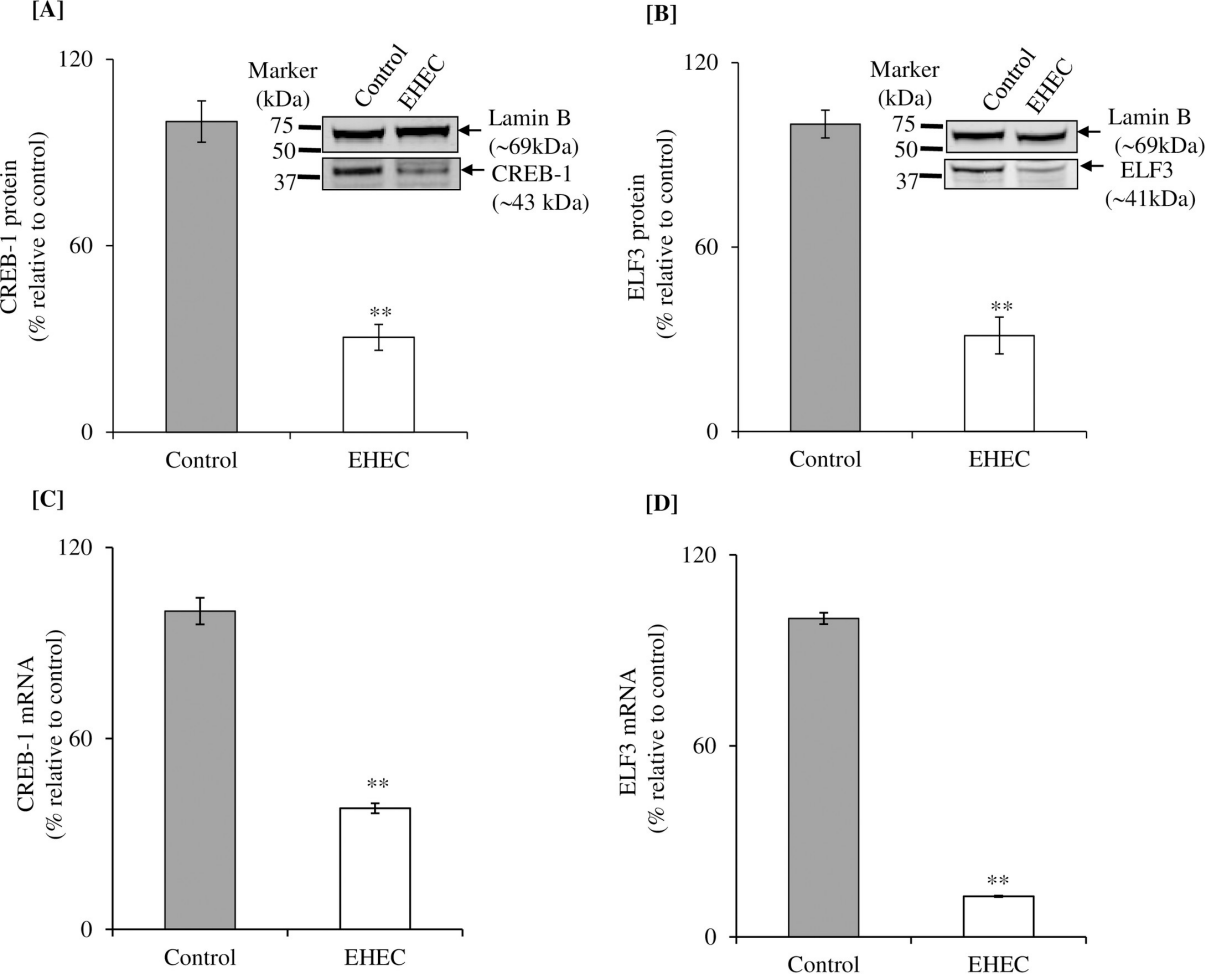
Effect of EHEC infection of NCM460 cells on level of expression of CREB-1 and ELF3 proteins and mRNAs. Levels of CREB-1 **[A]** and ELF3 **[B]** proteins expression were determined in NCM460 cells infected with EHEC (200 MOI for 5 h) by western blotting. Normalization of both the proteins expression was done relative to Lamin B. Levels of CREB-1 **[C]** and ELF3 **[D]** mRNA expression was determined in NCM460 cells infected with EHEC by RT-qPCR. Normalization of both the mRNAs expression was done by β-actin. Data are mean ± SE of 3 sets of independent experiments. **P < 0.01.

Previous findings from our laboratory have shown that the nuclear factors CREB-1 and ELF3 play important roles in driving basal promoter activity of the *SLC44A4* gene [24]. Thus, we also examined whether EHEC infection of NCM460 cells affects the expression of the CREB-1 and ELF3 transcription factors. The results showed that infecting NCM460 cells with EHEC leads to a significant (P < 0.01 for both) suppression in level of CREB-1 and ELF3 protein (Fig 4A & 4B), and mRNA (Fig 4C & 4D) expression.

### Role of ERK1/2 and NF-κB signaling pathways in mediating the inhibitory effect of EHEC on TPP uptake

**Fig 5:**
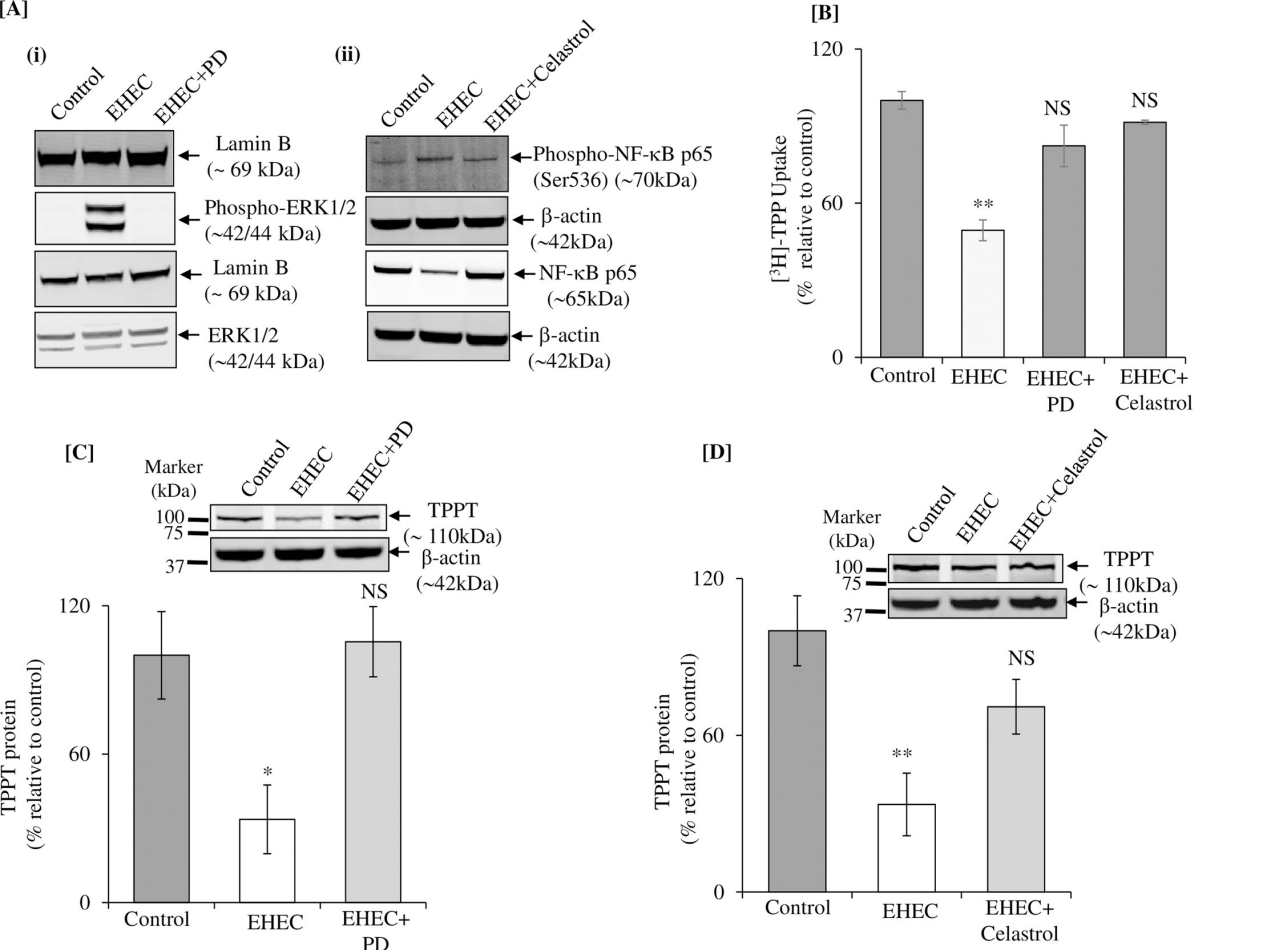
Role of ERK 1/2 and NF-κB signaling pathways in mediating the effect of EHEC infection of NCM460 cells on TPP uptake and on level of expression of TPPT. **[A]** Activation of ERK1/2 **(i)** and NF-κB **(ii)** in EHEC infected NCM460 cells. Cells were treated with EHEC and pretreated with 50 μM PD98059 (an ERK1/2 inhibitor) and 100 nM celastrol (an inhibitor of NF-kB). **[B]** Carrier-mediated [^3^H]-TPP uptake was examined in NCM460 cells that were pre-treated with PD98059 and celastrol for 1 h prior to EHEC (200 MOI for 5 h) infection. **[C and D]** Levels of TPPT protein expression in NCM460 cells that were pre-treated with PD98059 and celastrol prior to EHEC infection, respectively (western blotting). Data are mean ± SE of 4–6 different sets of independent experiments. **P < 0.01; *P < 0.05; NS-not significant; PD-PD98059 (inhibitor of ERK1/2) and celastrol (inhibitor of NF-κB).

It has been shown previously that EHEC infection of intestinal epithelial cells leads to a significant activation of the ERK 1/2 and NF-κB intracellular signaling pathways [36–38]. Thus, we first examined whether EHEC infection can activate ERK 1/2 and NF-κB signaling pathways in colonic NCM460 cells. The results showed that the phosphorylation of ERK1/2 and NF-κB was increased in EHEC-infected cells compared to uninfected control as well as to cells treated with ERK1/2 and NF-κB pathway inhibitors prior to EHEC infection (Fig 5A). These results indicate that EHEC infection in NCM460 cells activates these two pathways. Next, to determine whether these signaling pathways are involved in mediating the inhibition caused by EHEC in colonic TPP uptake and in level of expression of the TPPT, we used specific pharmacological inhibitors to block these pathways and examined the effect of such blocking on the inhibitory effect of EHEC on these parameters. We used the ERK 1/2 specific inhibitor PD98059 and the NF-κB specific inhibitor celastrol in our investigations [36–38]. The result showed that treatment of cells with PD98059 and celastrol to lead to significant (P < 0.01) abrogation in the inhibitory effect of EHEC on TPP uptake (Fig 5B) as well as on the level of expression of TPPT protein (Fig 5C & 5D). These findings suggest that ERK 1/2 and NF-kB signaling pathways are involved in mediating the effect of EHEC on the physiology and molecular biology of colonic TPP uptake process.

## Discussion

As mentioned earlier, studies from our laboratory have recently shown that the *SLC44A4* transport system is involved in the uptake of the microbiota-generated TPP and that expression of this transporter along the GI tract is restricted to the large intestine [21, 23]. We also shown that this uptake system is specific for TPP and does not transport the free form of vitamin B1, i. e., free thiamin. In addition, we have characterized different cell biology and regulatory aspects of this colonic TPPT system [20, 24, 25]. Currently, there is little known about the effect of external/ environmental factors on the function of this newly identified uptake system. In this study, we examined the effect of infection with the enteric pathogen EHEC on physiology/molecular biology of the colonic TPP uptake process. EHEC is a major enteric pathogen that causes foodborne diarrheal disease in the US; it affects approximately 75,000 individuals annually [39, 40]. Severe infection with this pathogen causes intestinal inflammation, hemorrhagic colitis, which could be followed by life-threatening hemolytic uremic syndrome [36, 37]. EHEC infection mainly impacts the colon and its effect is mediated via toxin-dependent and toxin-independent mechanisms [26, 27, 28]. Although EHEC has been shown to affect the physiology of ion transport in the colon [27, 40–43], its effect on uptake of micro-nutrients like water-soluble vitamins (including TPP) has not been examined. In this study, we employed as models: a well-established human-derived colonic epithelial NCM460 cells (for *in vitro* infection) and mice for (*in vivo* infection) to examine the effect of EHEC on colonic uptake of TPP. The results demonstrated that infection of NCM460 cells with live EHEC (but not heat-killed/boiled EHEC or with non-pathogenic *E*. *coli*) causes a marked inhibition in TPP uptake; no such inhibition in the substrate uptake was observed in cells treated with bacterial culture supernatant suggesting that the effect is not mediated via a secreted factor(s) (toxin) but rather it requires a direct contact of the bacterial with the colonic epithelial cells. Non-toxin mediated effect of EHEC infection on intestinal physiology has also been seen by others [33]. In addition, EHEC mutants with mutations in specific components of the T3SS system (Δtir-EHEC, ΔespF-EHEC and ΔescN-EHEC) also showed significant inhibition in TPP uptake, suggesting that these effector proteins are not involved in the EHEC mediated inhibition in TPP uptake. Similarly, *in vivo* infection of mice with EHEC led to a significant inhibition in colonic uptake of TPP. The inhibitory effect of EHEC on TPP uptake by human and mouse colonocytes was associated with a marked suppression in level of expression of TPPT protein and mRNA; the latter suggests that the effect is, at least in part, mediated at the level of transcription of the *SLC44A4* gene. This indeed appeared to be the case as activity of the full-length (as well as the minimal) *SLC44A4* promoter (transfected into NCM460 cells) were significantly reduced following infection with EHEC. Since the CREB-1 and ELF3 nuclear factors participate in driving promoter activity of the *SLC44A4* gene [24], we also examined whether EHEC infection of NCM460 cells affects expression of these factors. The results indeed showed that EHEC infection leads to a significant inhibition in the expression of both of these nuclear factors raising the possibility that they may be involved in mediating the inhibitory effect of EHEC on *SLC44A4* promoter activity.

Previous investigations have reported that the intracellular ERK 1/2 and NF-κB signaling pathways mediate the effects of EHEC on intestinal epithelial cells physiology [36–38]. Thus, we investigated whether these signaling pathways are also involved in mediating the inhibitory effect of EHEC on the physiology/molecular biology of the colonic TPP uptake process. For this, we used specific pharmacological inhibitors to suppress these pathways [36–38] and examined the effect of such treatment on the inhibitory effect of EHEC on TPP uptake and on expression of the TPPT protein in NCM460 cells. The results showed that inhibiting these signaling pathways to significantly reverse the inhibitory effect of EHEC on colonic TPP uptake and on level of TPPT protein expression. These findings suggest a role for these signaling pathways in mediating the inhibitory effect of EHEC on colonic TPP uptake and on level of expression of the TPPT.

## Conclusion

Results of these investigations show, for the first time, that EHEC infection inhibits colonic TPP uptake as well as level of expression of the involved transporter (i. e., TPPT), and that this inhibition is mediated, at least in part, at the level of *SLC44A4* transcription and may involve the ERK 1/2 and NF-κB signaling pathways.

## Supporting information

S1 File Relevant raw data and full western blot pictures. See the supplemental S1 File for uncropped full western gel and raw data for all the figures presented in this manuscript.
(PDF)
