# Supplementary material for: Enterohemorrhagic Escherichia coli infection inhibits colonic thiamin pyrophosphate uptake via transcriptional mechanism
Source: PLoS One. 2019 Oct 22;14(10):e0224234. doi: 10.1371/journal.pone.0224234 (PMC6804999; doi:10.1371/journal.pone.0224234)
Supplement: S1 File — See the supplemental S1 File for uncropped full western gel and raw data for all the figures presented in this manuscript. (PDF) [file pone.0224234.s001.pdf]

Validation of anti-TPPT Ab°

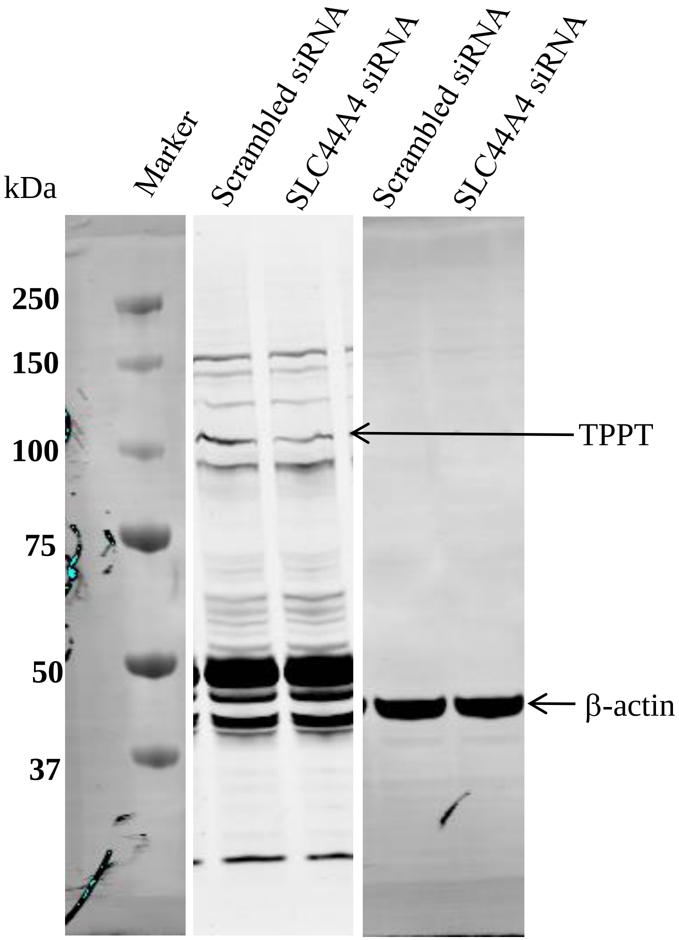

TPPT polyclonal antibodies were validated by knock down approach using SLC44A4 specific siRNA (Invitrogen, Cat. No. 10620318 and 10620319) in human colonic NCM460 cells.

Figure 1D

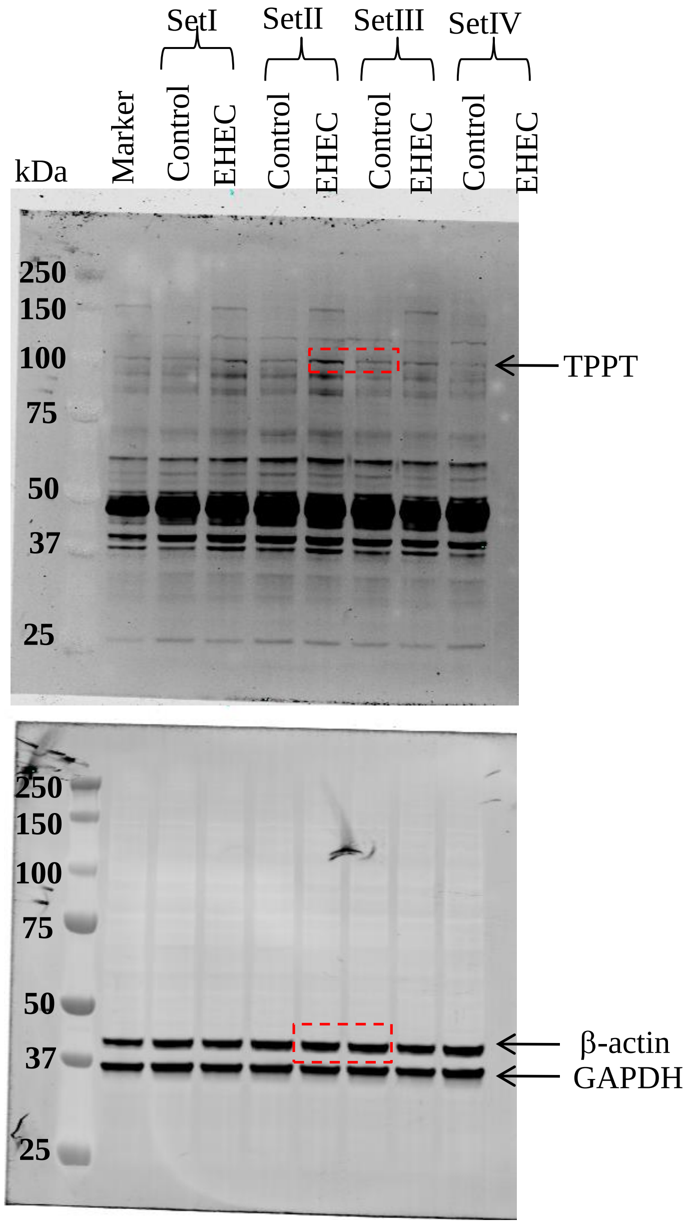

Red dotted box are presented as a representative bands

Figure 2B

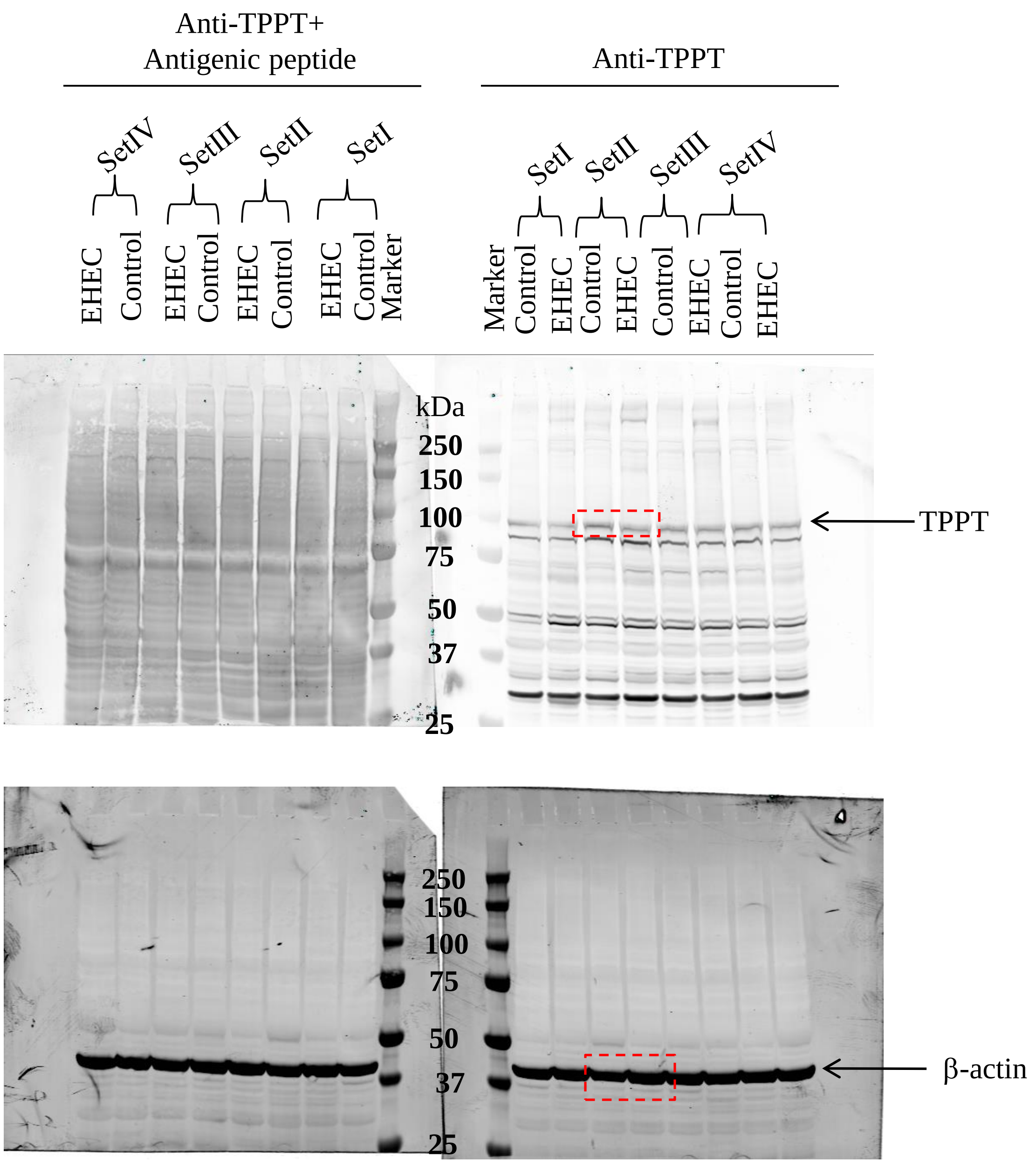

Red dotted box are presented as a representative bands

Figure 4B

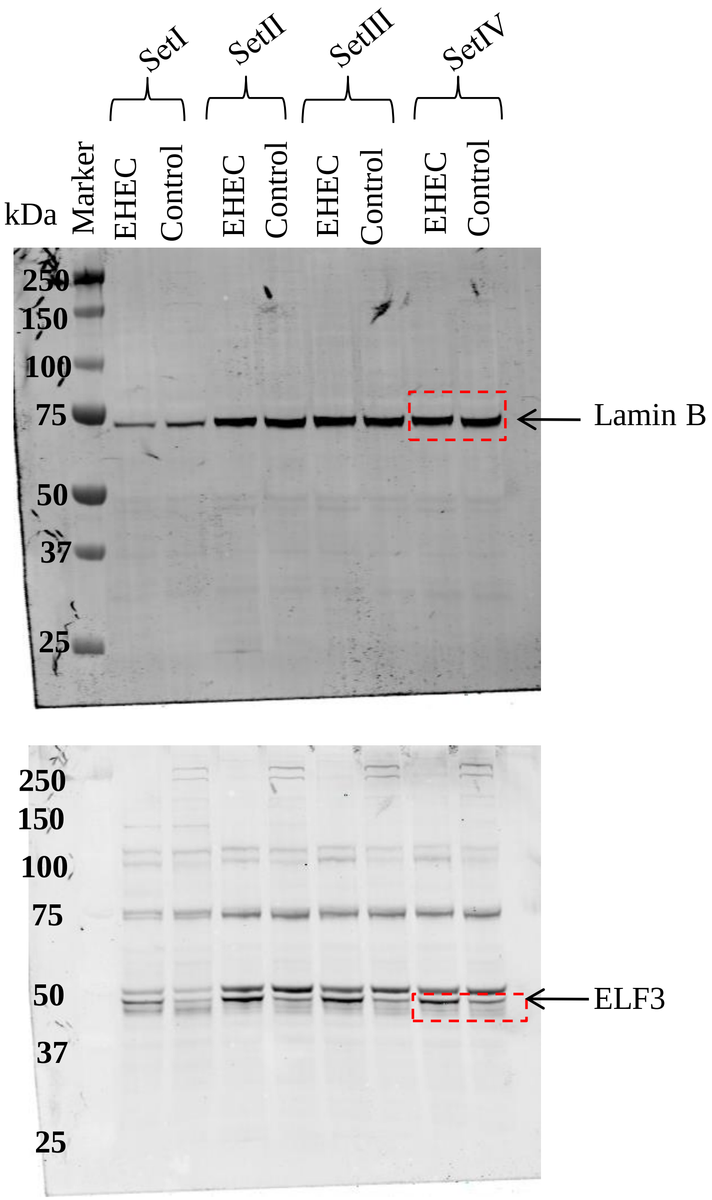

Figure 4A

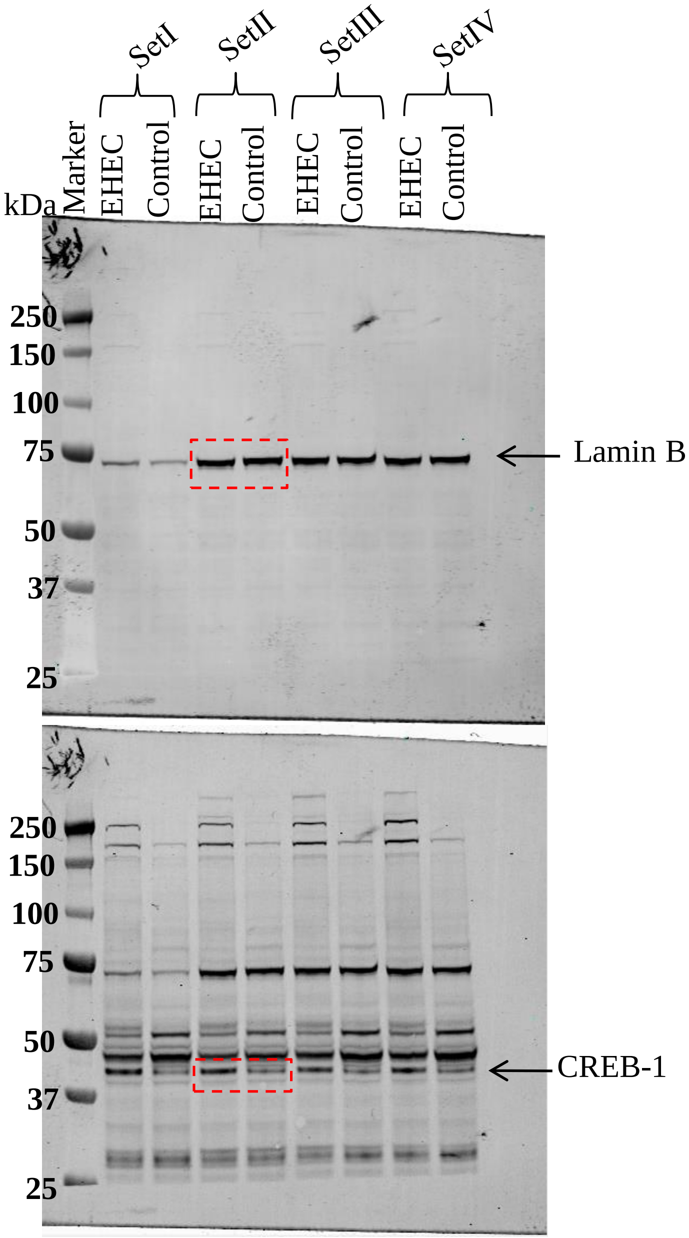

Red dotted box are presented as a representative bands

Figure 5A(i)

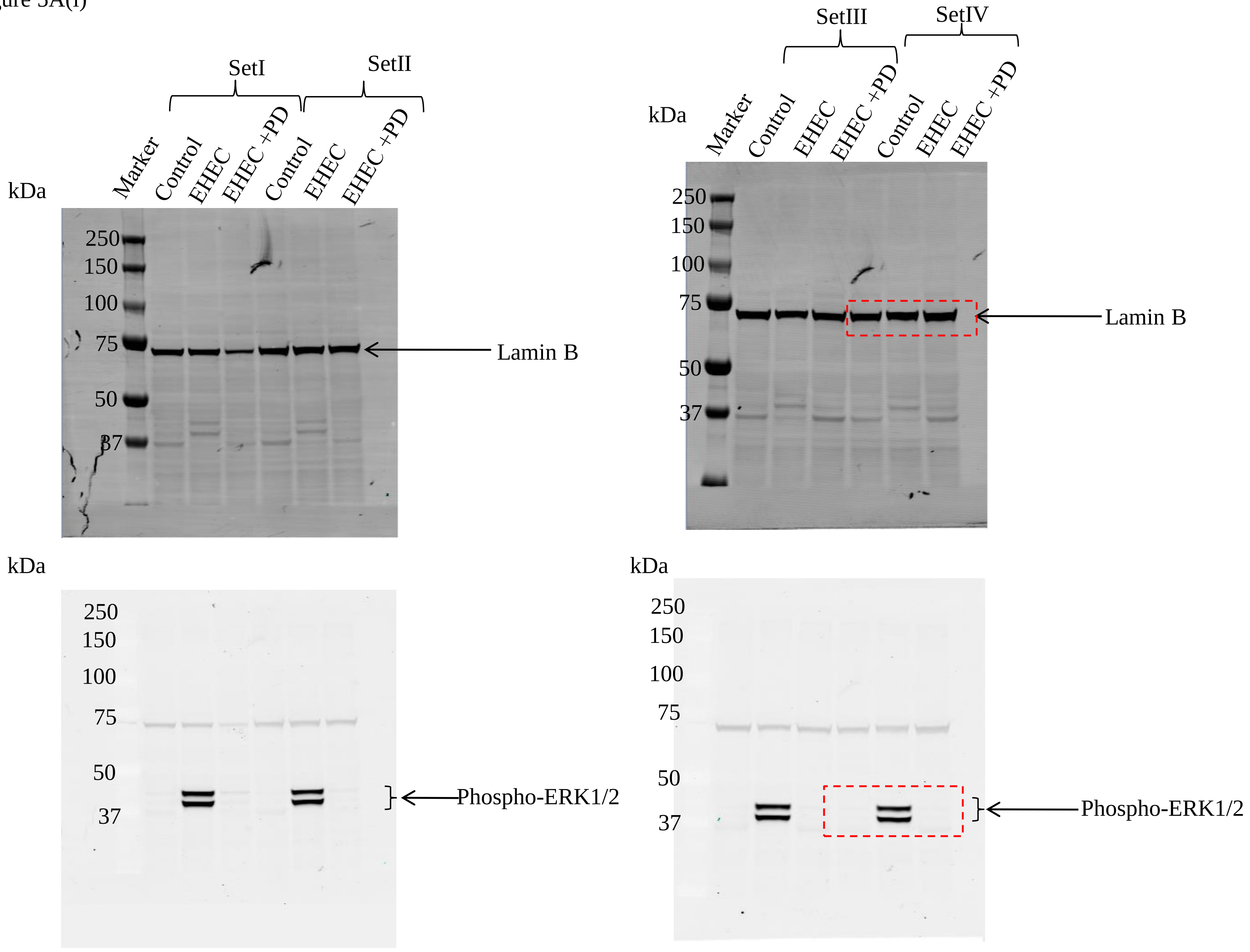
SetI
SetII
SetIII
SetIV
Marker
Control
EHEC
EHEC +PD
Control
EHEC
EHEC +PD
kDa
250
150
100
75
50
37
Lamin B
Phospho-ERK1/2

Figure 5A (i)

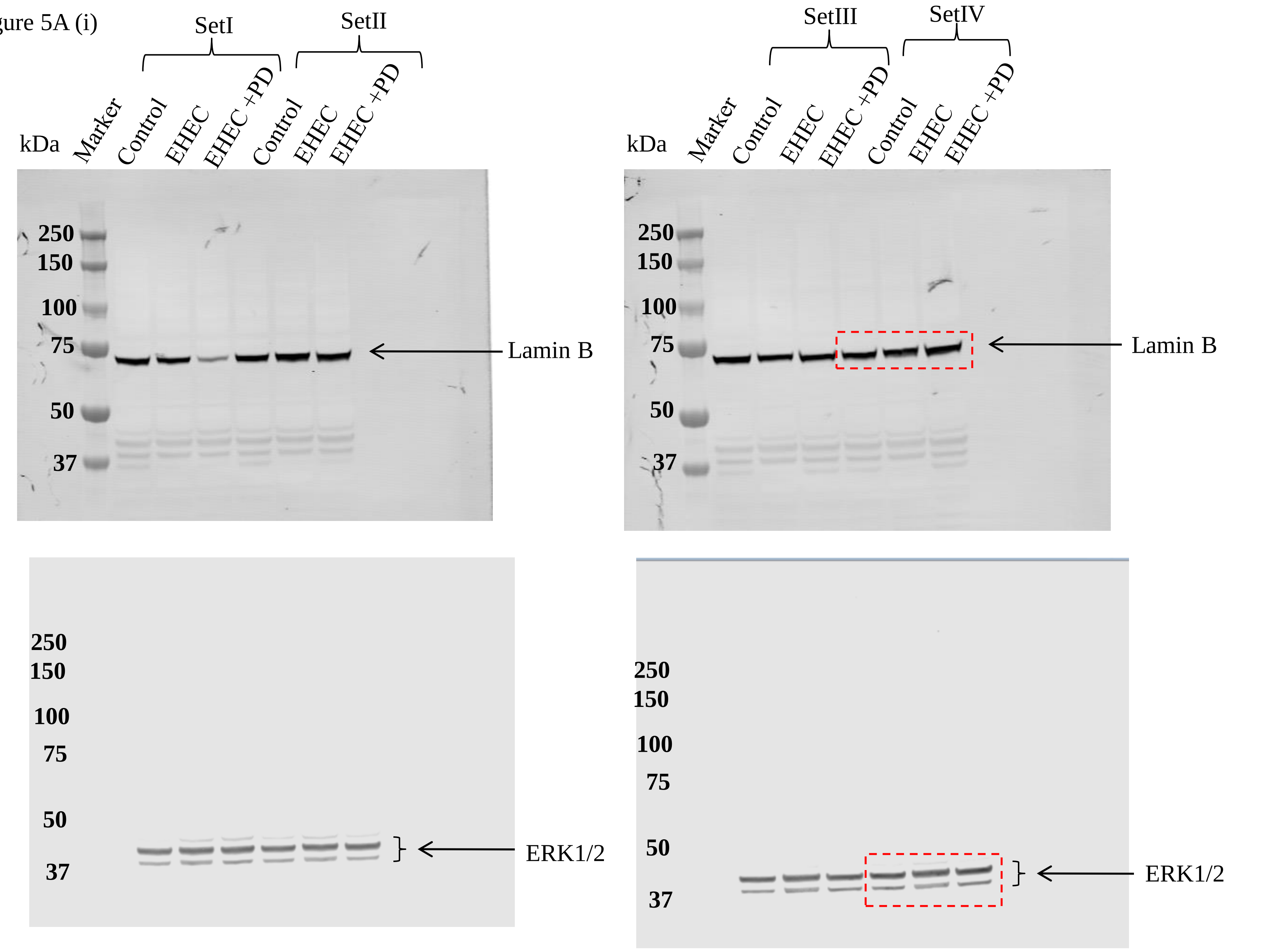

Red dotted box are presented as a representative bands

Figure 5A(ii)

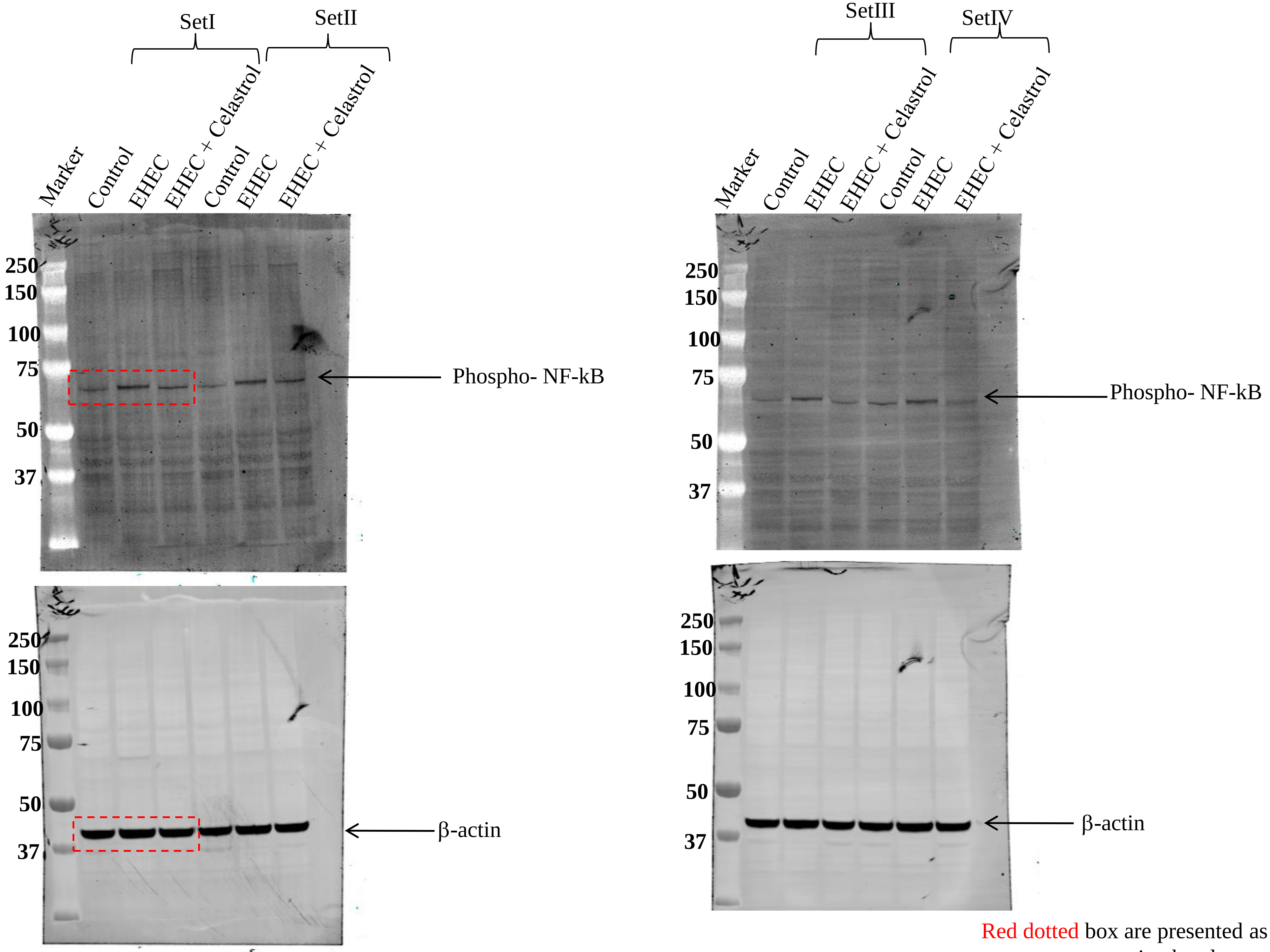

Red dotted box are presented as a representative bands

Figure 5A(ii)

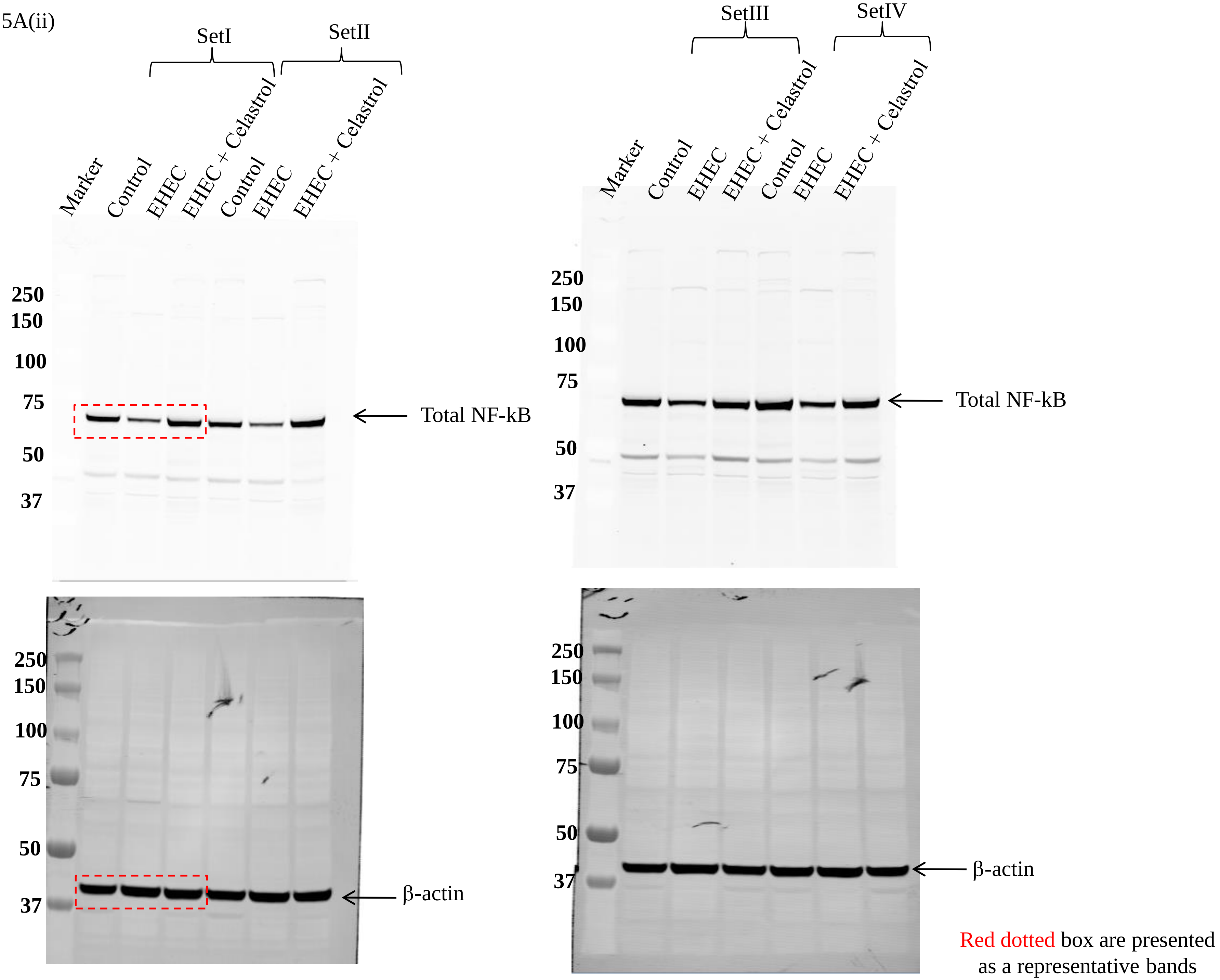

Red dotted box are presented as a representative bands

Figure 5C

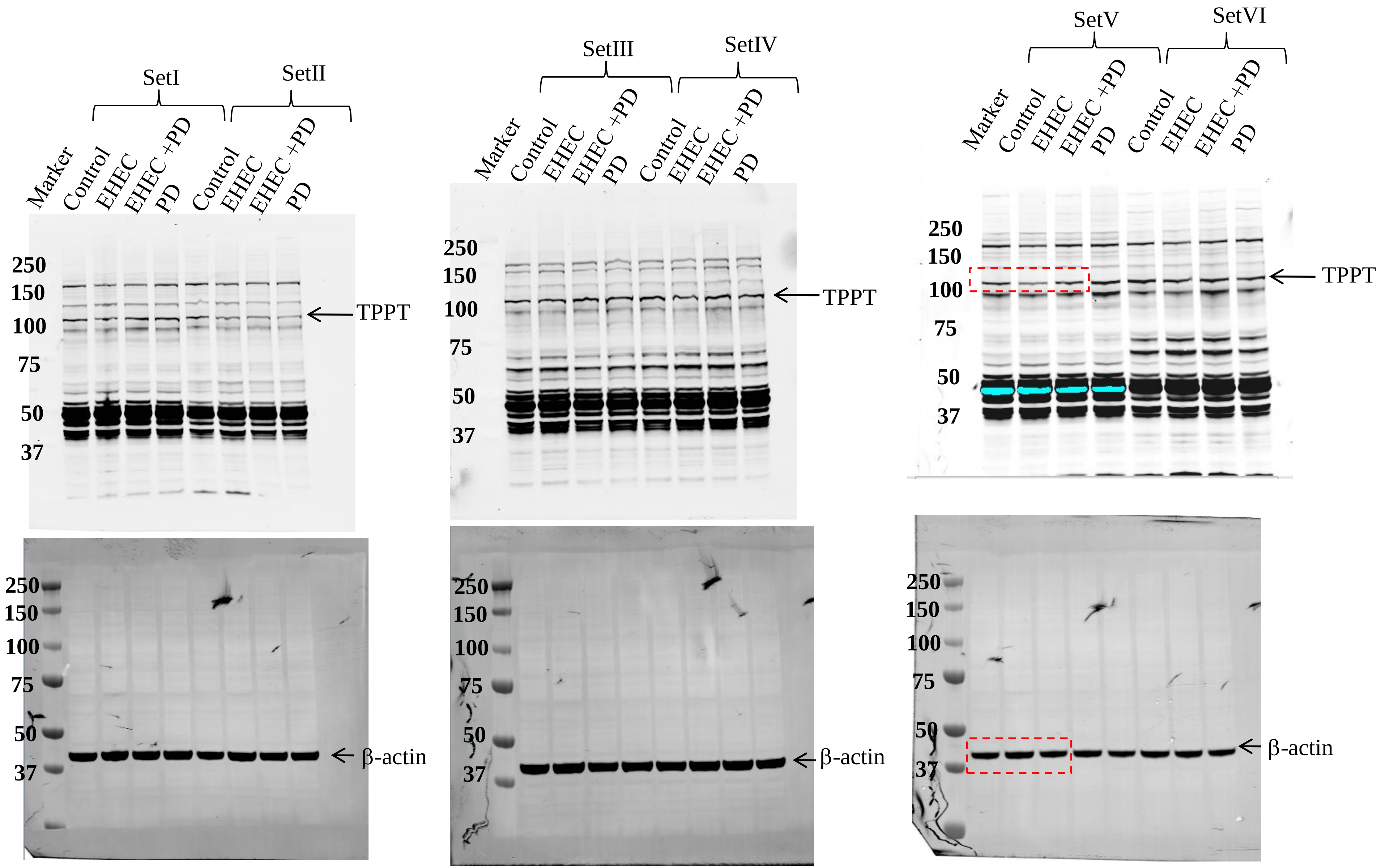

Red dotted box are presented as a representative bands

Figure 5D

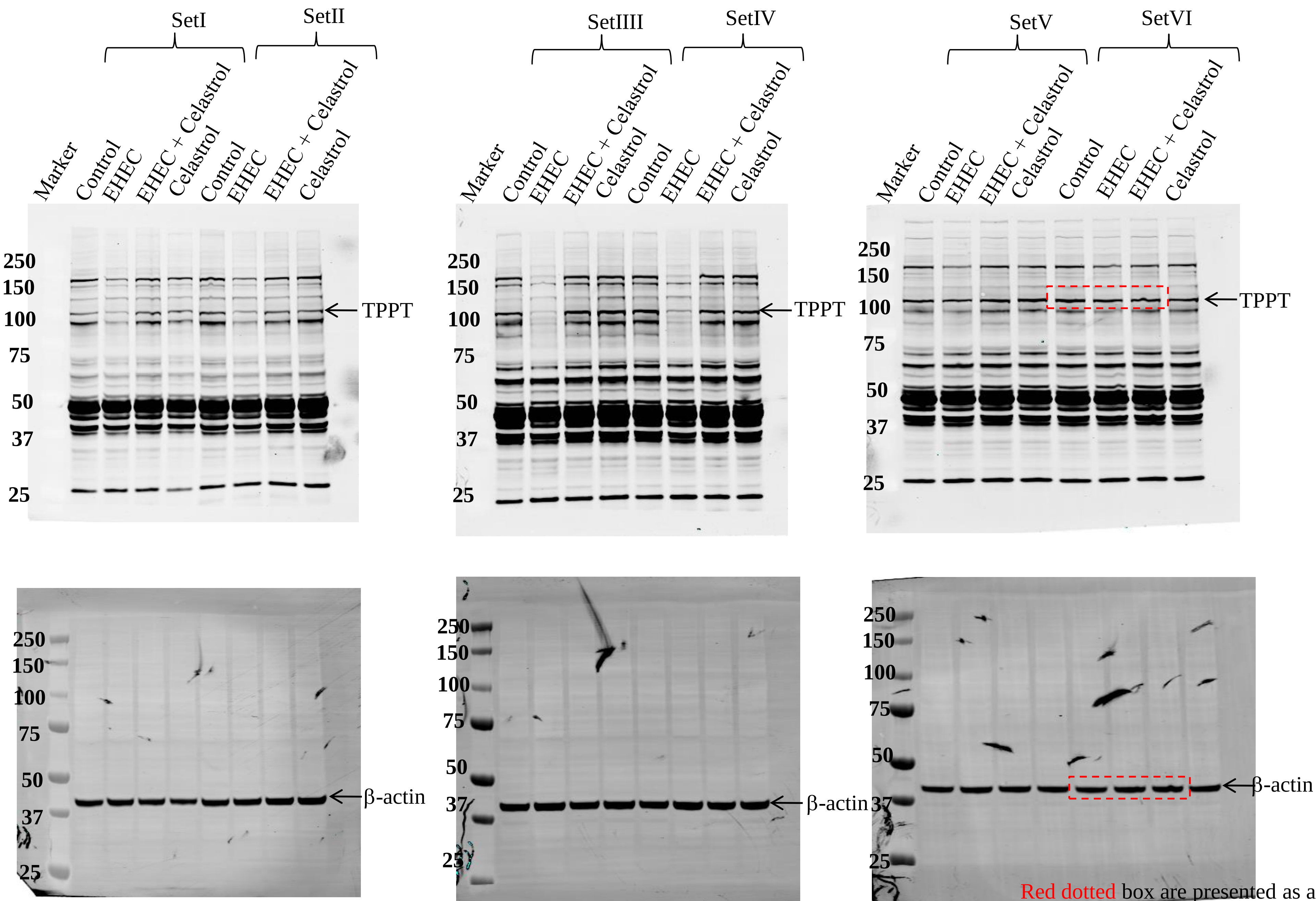

Red dotted box are presented as a representative bands

Figure 1A

in %	Set I	Set II	Set III	Mean	SE	P
Control	102.8	105.3	91.9	100.0	4.1	
EHEC (100 MOI)	62.3	57.7	87.9	69.3	9.4	0.04
EHEC (200 MOI)	57.7	45.3	50.3	51.1	3.6	0.001
HS4 (200 MOI)	91.2	90.9	112.9	98.3	7.3	0.85

Figure 1B

in %	Set I	Set II	Set III	Set IV	Mean	SE	P
Control	125.3	92.2	107.0	75.4	100	10.6	
EHEC	43.6	21.2	59.0	41.0	41.2	7.8	0.004243
Boiled EHEC	83.0	84.8	103.7	87.9	89.9	4.7	0.41736
EHEC Sup	98.0	99.3	102.4	96.5	99.1	1.3	0.934732

Figure 1C

in %	Set I	Set II	Set III	Mean	SE	P
Control	108.2	98.4	93.4	100	4.4	
EHEC	63.7	61.9	60.3	62.0	1.0	0.0010
Δtir	63.1	59.8	26.7	49.9	11.6	0.0156
ΔespF	59.5	53.7	33.2	48.8	8.0	0.0049
ΔescN	77.4	67.4	25.7	56.8	15.8	0.0581

Figure 1E

in %	Set 1	Set 2	Set 3	Mean	S. E	P
Control	96.3	99.8	103.8	100	2.2	
EHEC	32.4	54.6	30.0	39.0	7.8	0.001653

Figure 2A

in %	Mouse 1	Mouse 2	Mouse 3	Mean	SD	SE	P=
Control	76	117	107	100	17.3	10.0	
EHEC	30	39	35	35	3.9	2.2	0.006

Figure 2C

in %	Set-I	Set-II	Set-III	Set-IV	Mean	SD	SE	P=
Control	118	100	77	95	98	17	8.4	
EHEC	68	82	77	45	68	16	8.1	0.04448

Figure 3A

in %							
**Full length**	Set I	Set II	Set III	Set IV	mean	SE	p=
Control	91.9	92.4	115.8	48.3	100	7.9	
EHEC	72.8	78.3	63.5	80.9	71.5	4.3	0.033893

Figure 3B

in %							
**Minimal**	Set I	Set II	Set III	Set IV	mean	SE	p=
Control	91.2	119.8	96.7	92.3	100	6.7	
EHEC	64.2	60.9	62.7	43.6	57.9	4.8	0.002207

Figure 4C

in %	Set 1	Set 2	Set 3	Mean	SE	P
Control	99.74716	92.92035	107.3325	100	4.162346	
EHEC	38.02281	40.81633	35.33569	38.05828	1.582223	0.000118

Figure 4D

in %	Set 1	Set 2	Set 3	Mean	SE	P
Control	97.4	103.3	99.3	100	1.8	
EHEC	13.0	12.3	12.8	12.7	0.2	1.00E-04

Figure 5B

in %	Set I	Set II	Set III	Set IV	Set V	Set VI	Mean	SE	P
Control	93.4	110.7	95.9	93.4	110.7	95.9	100	3.4	
EHEC	37.4	51.7	59.1	37.4	51.7	59.1	49.4	4.0	2.35E-06
EHEC+PD98059	68.8	53.9	85.5	111.6	91.8	82.4	82.3	8.1	0.004451
EHEC+Celastrol	93.2	92.6	93.9	90.2	88.4	90.4	91.5	0.9	1.32E-06
